# Implications of aneuploidy for stem cell biology and brain therapeutics

**DOI:** 10.3389/fncel.2012.00036

**Published:** 2012-09-05

**Authors:** Sylvie Devalle, Rafaela C. Sartore, Bruna S. Paulsen, Helena L. Borges, Rodrigo A. P. Martins, Stevens K. Rehen

**Affiliations:** National Laboratory for Embryonic Stem Cells, Institute of Biomedical Sciences, Federal University of Rio de JaneiroRio de Janeiro, RJ, Brazil

**Keywords:** chromosomal instability, mosaicism, CNS, disease modeling, transplantation

## Abstract

Understanding the cellular basis of neurological disorders have advanced at a slow pace, especially due to the extreme invasiveness of brain biopsying and limitations of cell lines and animal models that have been used. Since the derivation of pluripotent stem cells (PSCs), a novel source of cells for regenerative medicine and disease modeling has become available, holding great potential for the neurology field. However, safety for therapy and accurateness for modeling have been a matter of intense debate, considering that genomic instability, including the gain and loss of chromosomes (aneuploidy), has been repeatedly observed in those cells. Despite the fact that recent reports have described some degree of aneuploidy as being normal during neuronal differentiation and present in healthy human brains, this phenomenon is particularly controversial since it has traditionally been associated with cancer and disabling syndromes. It is therefore necessary to appreciate, to which extent, aneuploid pluripotent stem cells are suitable for regenerative medicine and neurological modeling and also the limits that separate constitutive from disease-related aneuploidy. In this review, recent findings regarding chromosomal instability in PSCs and within the brain will be discussed.

## Introduction

Mitotic neural progenitor cells (NPCs) are frequently aneuploid (Rehen et al., 2001) and, albeit neurogenesis is accompanied by massive cell death (Blaschke et al., 1996) which may reduce brain aneuploidy (Yurov et al., 2005; Mosch et al., 2007), a significant proportion remains and generates long-life lasting mature aneuploid neurons (Rehen et al., 2005) capable of integrating to the brain circuitry (Kingsbury et al., 2005). The biological effects of aneuploidy in the brain are still a matter of speculation, but it possibly serves as a diversity generation mechanism (Rehen et al., 2001, 2005; Kingsbury et al., 2006; Iourov et al., 2006). Loss of heterozygosity (LOH) produced by chromosomal loss can alter gene expression without affecting cell proliferation and survival (Kaushal et al., 2003). In a neuronal network, connection of aneuploid cells to euploid cells is expected to confer extra variability contributing to interpersonal uniqueness (Kingsbury et al., 2005; Muotri and Gage, 2006), however, a narrow boundary between aneuploidy as a diversifier agent in healthy brain and as a prompter of diseases may exist since this phenomenon has been repeatedly observed in pathological conditions.

Although neurological disturbances have been known for centuries, they remain poorly understood, partially due to limitation of available experimental approaches (*post-mortem* brains, immortalized human cells or animal models), and treatments are still unsatisfactory (Lukiw, 2012; Miyamoto et al., 2012). Therefore, development and validation of novel *in vitro* models may accelerate knowledge directed to neurological disturbances. Pluripotent stem cells (PSCs) have the potential to self-renew and generate cells from the three germ layers, been largely considered as a promising source for modeling and drug screening. The main PSCs are embryonic stem cells (ESCs), which are derived from the inner cell mass of blastocyst, and induced pluripotent stem cells (iPSCs), generated through induction of pluripotency factors expression in somatic cells (Takahashi et al., 2007). While ESCs represent the natural PSCs prototype, iPSCs have the advantage to overcome ethical and (in principle) incompatibility issues inherent to its embryonic origin as well as to provide a source for disease modeling that cannot be tracked by preimplantation genetic diagnosis (PGD).

Many diseases are associated to genetic components and various alleles can predispose to the same pathological outcome. Patient-specific somatic cells reprogrammed into iPSCs preserve its particular genetic background, providing an alternative to study diseases in a non-invasive manner, without prior knowledge of disorders-associated genes. Moreover, they possess the unique capacity to recapitulate development in an embryo-like fashion, representing an excellent source to study neurogenesis and neurodevelopmental diseases. Indeed, several studies have described cells differentiated from iPSCs able to recapitulate many aspects of distinct disorders, demonstrating their applicability for disease modeling (Ebben et al., 2011; Grskovic et al., 2011; Kunkanjanawan et al., 2011; Saha and Hurlbut, 2011; Tiscornia et al., 2011; Oh et al., 2012). However, to serve as a source for cell therapy and to model diseases reliably, PSCs need to be safe and preserve phenotypic aspects observed during development. The description of genomic modifications in PSCs, especially in the artificially generated iPSCs, gave rise to an intense debate on their usefulness (Panopoulos et al., 2011; Pera, 2011; Ross et al., 2011).

Genomic modifications can result in genomic instability and are measured at different resolution levels. Such modifications are depicted as aneuploidy, chromosome rearrangements, copy number variation (CNV) and single nucleotide polymorphism (SNP). Aneuploidy involves loss or gain of one or more chromosomes compared to the original species' set. In contrast, other modifications mentioned are confined to portions of chromosomes. More specifically, while rearrangements spatially reorganize genes and/or their regulatory elements, CNVs consist of duplication or deletion of DNA portions, whereas SNPs are single nucleotide's alterations. Such changes can have drastic effects for cells, altering gene dosage and integrity.

Genome alterations (particularly aneuploidy) have been largely correlated with cancer, malformation, miscarriage and other pathologies (Duesberg et al., 2006; Conrad et al., 2010; Lebedev, 2011; Coschi and Dick, 2012), although their participation in evolutionary processes is well recognized (Cooper et al., 2004; Nguyen et al., 2008; Pavelka et al., 2010; Stenberg and Larsson, 2011). As mentioned previously, such variations have been suggested to contribute to diversity in the healthy brain (Kingsbury et al., 2005; Rehen et al., 2005; Westra et al., 2008) and evidences also indicate that PSCs are not homogeneously euploid, which suggest that chromosomal mosaicism is inherent to these cells (Peterson et al., 2011), probably as consequence of their singular cell cycle (White and Dalton, 2005).

Aneuploidy as a natural phenomenon in healthy individuals is a relatively new concept. Up to now, only a few tissues have been demonstrated to tolerate aneuploidy, and its usual association with pathogenic contexts generates the need to distinguish normal from disease-related gain and loss of chromosomes. Given the existence of aneuploidy in PSCs, their suitability as a source for cell therapy and modeling will be considered. Moreover, a brief description of aneuploidy in mental disorders will be undertaken in an attempt to clarify the boundaries separating normal from disease-related chromosomal mosaicism.

## Aneuploidy in mental disorders

From all genomic modifications, ploidy is particularly drastic since gains or losses of whole chromosomes abruptly alter the dosage of hundreds of genes in a cell, leading to possible imbalances in critical proteins. LOH, described as the change of a heterozygous state to a homozygous state, can arise after loss of a whole chromosome and have severe effects. Monosomy and trisomy of almost all chromosomes in the embryo are lethal. Trisomy 13, 18, and 21 are the only non-sexual-chromosome aneuploidies that allow full term pregnancy, and severity of phenotypes depends on the incidence of the abnormality among their cells. Interestingly, these chromosomes contain the fewest protein-coding genes (Torres et al., 2008). Therefore, different types and frequencies of mosaic aneuploidy in individuals might have more or less tolerable effects for cell function and adaptation under stress conditions.

Mosaic aneuploidy has been described as a normal occurrence in adult and developing brains (Rehen et al., 2005; Yurov et al., 2005, 2007a) but it has most traditionally been associated with pathologies. It has therefore been postulated that aneuploidy may be harmless or detrimental to proper functioning of CNS depending on its level. In fact, a 2 fold random aneuploidy and a 4 fold specific chromosome 21 aneuploidy increase was reported in ataxia telangiectasia's (AT) and Alzheimer disease's (AD) patients brains, respectively (Iourov et al., 2009b). As shown in Table 1, AT brain aneuploidy encompasses both gain and loss of almost all chromosomes, whereas AD brains display preferential unbalance of chromosome 21 and chromosome 17 (mostly gain). Importantly, amyloid precursor gene, responsible for Aβ peptide production, is localized at chromosome 21. Analogously, tau gene, which encodes a component of neurofibrillary tangles in AD, is located on chromosome 17, reinforcing the contribution of such excessive chromosomal dosages for AD etiology.

**Table 1:** **Aneuploidy in healthy and neurological disordered brains**.

**Chromosomes**	**% Aneuploidy (loss, gain)**			
	**Healthy brain**	**AT**	**AD**	**Schizo**
1	0.5 (0.3, 0.2)	2.8 (1.7, 1.1)	0.7 (0.3, 0.4)	1.8 (0.9, 0.9)
7	0.7 (0.3, 0.4)	1.5 (0.6, 0.8)	NA	NA
8	1.0 (0.3, 0.7)	2.8 (0.8, 2.0)	NA	NA
9	1.2 (0.5, 0.7)	1.3 (0.6, 0.7)	NA	NA
11	0.8 (0.4, 0.3)	3.1 (1.5, 1.5)	1.1 (0.7, 0.4)	NA
16	0.7 (0.1, 0.6)	2.8 (0.4, 2.5)	NA	NA
17	2.2 (0.4, 1.8)	2.4 (1.2, 1.2)	7.7 (0.5, 7.2)	NA
18	0.9 (0.5, 0.4)	2.5 (1.2, 1.2)	1.0 (0.5, 0.4)	0.5 (NR, 0.5)
21	2.5 (1.3, 1.3)	NA	10.7 (4.1, 6.6)	NA
X	0.7 (0.3, 0.4)	1.8 (0.3, 1.5)	1.9 (0.7, 1.2)	1.2 (NR, 1.2)
Y	0.2 (0.1, 0.1)	0.6 (0.0, 0.6)	NA	NA

NR, Not reported; NA, Not available; AT, Ataxia telangiectasia; AD, Alzheimer's disease; schizophrenia (schizo). Mean aneuploidy was calculated based on Rehen et al. (2005); Mosch et al. (2007); Yurov et al. (2008); Iourov et al. (2009b) for healthy brain, (Iourov et al., 2009a,b) for AT, (Mosch et al., 2007; Iourov et al., 2009b) for AD and (Yurov et al., 2001, 2008) for schizophrenia.

Schizophrenic patients were also described to have X-aneuploidy in a frequency 4–6 higher than normal subjects in blood cells (Delisi et al., 1994; Bassett et al., 2000). Additionally, chromosome 1 aneuploidy is three times higher in schizophrenic brains than in normal brains (Table 1). Notably, specific genes such as *DISC1* (disrupted in schizophrenia 1) and *neuregulin 1*, localized at chromosome 1, have been associated with the disorder (Sullivan, 2008). Moreover, a cohort of autistic children was identified to display increased frequency of aneuploidies involving chromosomes 9, 15, 16, 18, and X in peripheral blood lymphocytes (Yurov et al., 2007b). Nonetheless, only few patients show augmented levels of chromosomal mosaicism in the brain, suggesting that aneuploidy may just contribute to a certain proportion of cases.

Another argument favoring the “aneuploidy dosage” hypothesis is based on the fact that Down's syndrome individuals with distinct levels of trisomic 21 cells vary from normal development (Verresen et al., 1964; Kohn et al., 1970) to mild (Ringman et al., 2008) or severe developmental delay (Richards, 1969). This indicates that frequency and type of aneuploidy might dictate whether a tissue will stay healthy or become diseased.

**Figure 1:**
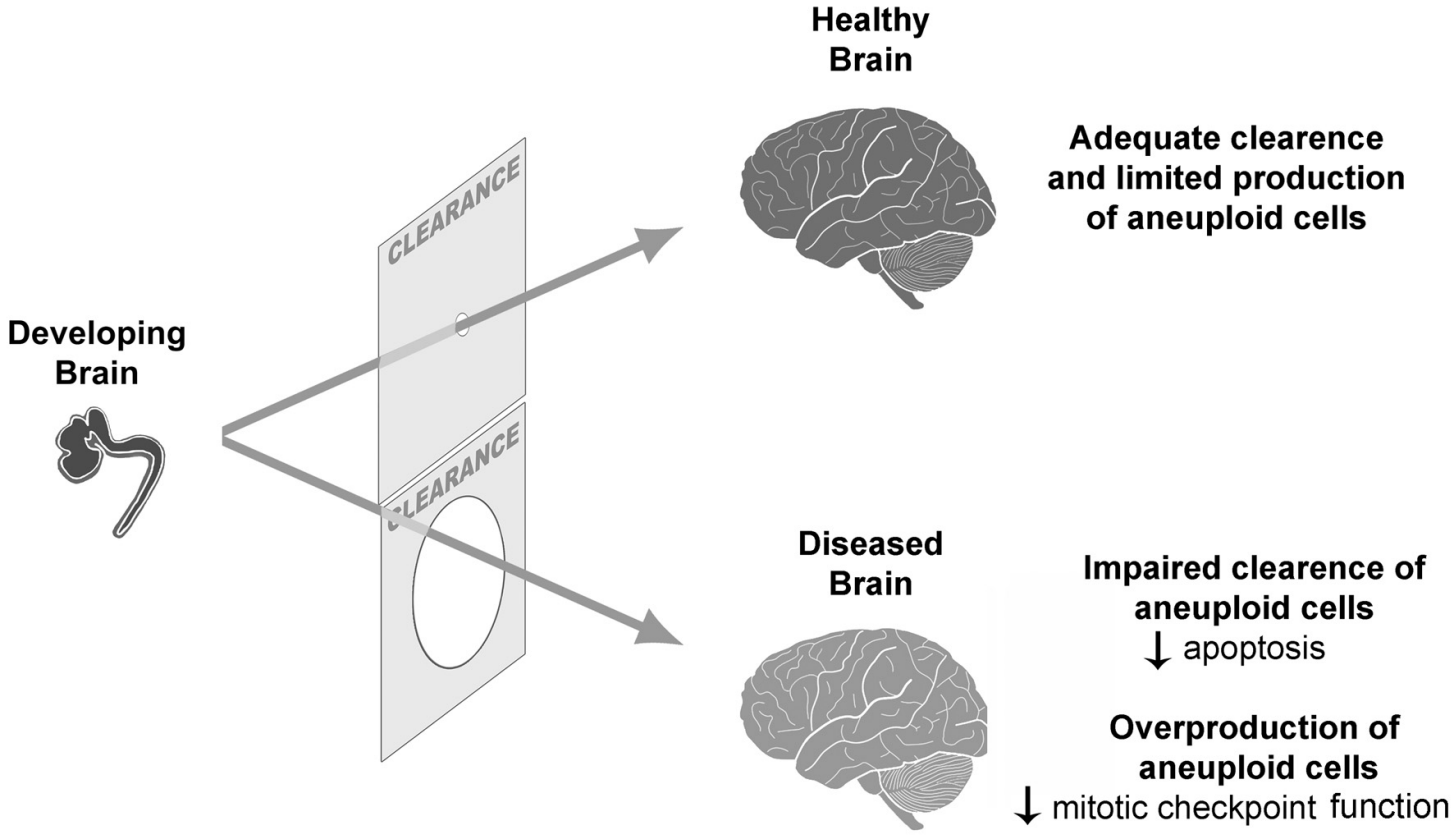
**Aneuploidy during neurogenesis is followed by a clearance of excessive aneuploid cells.** If there is a defective clearance or an overproduction of aneuploid cells, an increased frequency of aneuploidy might contribute to CNS pathogenesis.

But how could, then, the normal limit of tolerable aneuploidy be surpassed? In a normal scenario of neurogenesis, both overproduction and clearance of neural progenitors occur (Blaschke et al., 1996) concomitantly with aneuploidy (Sartore et al., 2011). While neutral and/or benign aneuploidies would persist, giving rise to genetic mosaicism in the brain, some results have suggested that detrimental aneuploidies would be cleared (Rehen et al., 2001; Kaushal et al., 2003). Considering this situation, an inefficient clearance could lead to an excessive accumulation of aneuploid cells in some neurological diseases (Figure 1). In *Atm* ^−/−^ mice, gain and loss of chromosomes are increased in embryonic NPCs and adult cerebral cortex, suggesting that clearance of aneuploid NPCs by apoptosis is deficient in these mice. Likewise, as mentioned, AT human brains present elevated aneuploidy (Iourov et al., 2009a). Another example of a neurological disorder with excessive aneuploidy is mosaic variegated aneuploidy syndrome (MVA), a disease that can be caused by *BUB1B* mutations, which leads to impaired mitotic checkpoint and aneuploidy (Bohers et al., 2008; Suijkerbuijk et al., 2010).

Identifying chromosomal abnormalities associated with neurological disorders may help locating and predicting genes involved in those complex diseases. However, the analysis of gain and loss of chromosomes in *post-mortem* brain may pose some limitations, as it resembles the final-stage of the disease, leading to misinterpretations due to secondary causes, such as chronic use of an antipsychotic or aging itself. Likewise, animal models cannot correctly predict human genes participating in diseases due to lack of architectural chromosomes equivalence. In this regard, models such as iPSCs, which allow to recapitulate neurodevelopmental stages and correctly track genetic changes, will certainly have greater application in understanding the etiology of unknown-causative brain disorders.

## Aneuploidy in pluripotent stem cells

It is now clear that contrarily to what was believed after its derivation, many PSCs exhibit some degree of aneuploidy (Peterson et al., 2011). Even though first considered solely as a hurdle for the organisms, aneuploidy is starting to be considered as a normal phenomenon in certain cases. Indeed, random mosaic aneuploidy is frequent in embryos at cleavage stage (Munne et al., 1994; Vanneste et al., 2009; Yurov et al., 2009; Iourov et al., 2010; Robberecht et al., 2010; Mantzouratou and Delhanty, 2011; Van Echten-Arends et al., 2011; Nagaoka et al., 2012; Vanneste et al., 2012) independently of maternal age (Delhanty et al., 1997; Baart et al., 2006). Despite the fact that many of those mosaic aneuploid embryos result in abortion (Warburton et al., 1978; Philipp et al., 2003; Lebedev, 2011), later-phase euploid embryos previously diagnosed as aneuploid are frequent (Li et al., 2005), which indicates their ability to cope with this phenomenon. Actually, because the majority of cells in the preimplantation embryo give rise to the placenta, most aneuploid cells turn out to be located in this structure, limiting aneuploidy's contribution to the fetal body. A possibility is that in the ectotrophoblast destined to become the placenta, early aneuploidy generation would help to enhance invasiveness, assisting embryo to implant (Weier et al., 2006), which is in agreement with its natural occurrence during embryogenesis. In fact, chromosomal mosaicism might even have been underestimated since PGD only analyses a few chromosomes from one of a total of 6–10 blastomeres and several works corroborate the deficiency of PGD single-blastomere analysis in detecting chromosomal mosaicism (Wells and Delhanty, 2000; Baart et al., 2006; Coulam et al., 2007; Ambartsumyan and Clark, 2008; Vanneste et al., 2009).

**Figure 2:**
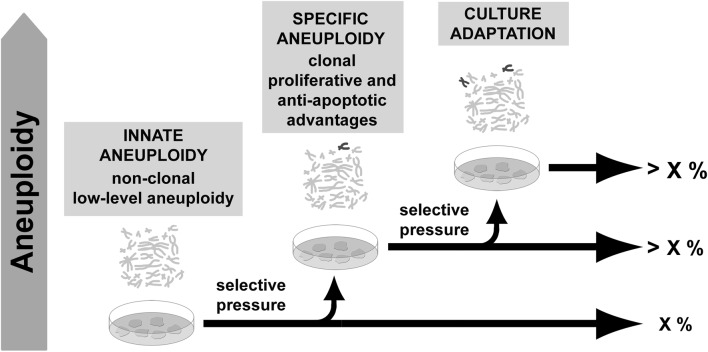
**Aneuploidy in pluripotent stem cells.** Low-level aneuploidy affecting multiple chromosomes may be a residual feature of cells from the inner cell mass. In culture, cells are under selective environment and some kinds of aneuploidy may benefit from improved self-renewal, proliferation or anti-apoptotic properties. As a result, cells carrying such aneuploidy may overcome the culture.

Despite increasing evidence of a normal occurrence of aneuploidy before derivation, not all PSCs aneuploidies seem to be products of *in vivo* selection. Indeed, since PSCs derivation, clonal genomic modifications were described to contribute to culture adaptation constituting a limitation for their use. Studies indicate that mechanical disaggregation of colonies tends to preserve chromosomal stability (Mitalipova et al., 2005; Catalina et al., 2008; Rashid et al., 2010) probably due to preservation of cell-cell contact and paracrine signaling necessary for their survival (Moogk et al., 2010). Absence of such signals during single-cell splitting could represent a pressure favoring the ascendance of aneuploid cells adapted to the new situation. Increased passaging was also correlated with genomic modifications (Enver et al., 2005; Maitra et al., 2005; Baker et al., 2007; Mayshar et al., 2010; Laurent et al., 2011; Ross et al., 2011), while low levels of O_2,_ compatible with *in vivo* condition, were found to favor genome integrity in ESCs cultures (Forsyth et al., 2006; Lim et al., 2011). Such descriptions are compatible with cell adaptation to stressful and unnatural conditions (long term self-renewal and high oxidative stress *in vitro*), once such aneuploidies involve recurrent gain of the same chromosomes and tend to overcome cell culture (Amps et al., 2011). Contrastingly, no correlation could be established between basal aneuploidy presence and any particular culture condition, including media, supplements or substrate as reported in a recent study from Peterson and coworkers (2011). This suggests that intrinsic conditions, other than culture conditions *per se*, contribute to aneuploidy as well (Figure 2).

The reason why all PSCs were previously believed to be homogeneously euploid probably relies on both reduced number of metaphases analyzed (Lin et al., 2007; Mai et al., 2007; Itoh et al., 2011) and acceptance that hypoploidy was a technical artifact as stated by standard cytogenetic books (Barch et al., 1997; Meisner and Johnson, 2008; Zheng and Dean, 2009). Many papers reporting euploid genomes have outsourced karyotyping (Lee et al., 2009; Chamberlain et al., 2010; Ananiev et al., 2011; Mitne-Neto et al., 2011; Seibler et al., 2011; Sheridan et al., 2011), counted only 20–50 (Lin et al., 2007; Mai et al., 2007; Itoh et al., 2011) or omitted the number of counted cells (Ye et al., 2009; Carvajal-Vergara et al., 2010; Cheung et al., 2011; Jin et al., 2011; Liu et al., 2011; Tolar et al., 2011), probably underscoring low-level mosaic aneuploidies, since at least 100 metaphases were counted by Peterson and colleagues to detected 3% aneuploidy (Peterson et al., 2011). Moreover, 18–35% of cultured cells from 6 distinct PSCs lines displayed random aneuploidy affecting different chromosomes at low rates (Peterson et al., 2011). The absence of specific recurrence suggests that low-level mosaic aneuploidy must not confer selective advantage to cells, being therefore unrelated to culture adaptation. Furthermore, it is noteworthy that, like in healthy liver and brain, chromosome losses account for most aneuploidies observed in PSCs (Peterson et al., 2011), contrasting with a predominance of chromosomal gains in mental disorders and cancer. Importantly, results were confirmed by FISH (interphase nuclei), which discards the possibility of technical artifacts to explain loss of chromosomes. Besides, constitutive PSCs mosaic aneuploidy is consistent with and could help explaining the observed phenotypic and functional heterogeneity within cultures (Chambers et al., 2007; Singh et al., 2007; Graf and Stadtfeld, 2008; Hayashi et al., 2008; Chin et al., 2009; Hough et al., 2009).

*In vivo*, chromosomal mosaicism might be a diversifying agent conferring an advantage for embryo survival in certain conditions (i.e., malnutrition during pregnancy, atrophied endometrium, women under medication, etc). Therefore, it is probable that low-level stochastic chromosomal aneuploidy (especially losses) is well tolerated and contributes to enhanced capacity to overcome environmental changes and to respond contrastingly (self-renewal or specialization in distinct cell types) to differentiation stimuli. On the other hand, gain of chromosomes seem to have an unfavorable outcome in somatic cells since they tend to disappear following differentiation (Mantel et al., 2007), decrease in later-stage embryos (Evsikov and Verlinsky, 1998), be confined to placenta (Kalousek, 1993; Gonzalez-Merino et al., 2003) or, in worst cases, result in abortion or malfunctioning. In addition, chromosomal mosaicism has also been described within the developing human brain (Yurov et al., 2007a) with no clear correlation with neurological diseases.

## Are induced pluripotent stem cells prone to aneuploidy?

Somatic cell origin of iPSCs raised the concern that they would display more genome modifications than ESCs, as cells from older individuals, subjected to environmental mutagenic influences, would have greater chance to accumulate DNA abnormalities. Although iPSCs harboring DNA changes originated in somatic cell progenitors have been described (Mayshar et al., 2010; Gore et al., 2011; Ji et al., 2012), the inner cell mass—as the origin of ESCs—commonly exhibits aneuploidy as well (Munne et al., 1994; Vanneste et al., 2009; Mantzouratou and Delhanty, 2011). It is unclear whether the frequency of genomic modifications in somatic cells is higher than in the inner cell mass, although in mice, somatic cells' mutation-rate seems to be superior to that of ESCs (10^−4^ compared to 10^−6^, respectively) (Cervantes et al., 2002).

Reprogramming methods used to create iPSCs has also been a matter of concern. Lenti or retrovirus-derived iPSCs were generally considered as genomic-instability-prone, since random DNA integration of pluripotency factors could disrupt genes involved in tasks such as DNA repair and cell cycle. Undoubtedly, reprogramming represents a stress to cells, which may facilitate genomic abnormalities to arise. However, since genomic instability is seen in reprogrammed cells regardless of employed reprogramming method, it might rather reflect the enforced phenotype change imposed to somatic cells than the chosen technique (Gore et al., 2011; Taapken et al., 2011). Paired comparative studies of iPSCs reprogrammed from the same somatic cell type, using different approaches, are required in order to clarify the contribution of reprogramming methods to genome instability.

Qualitatively, only few chromosomal changes seem unique to either ESCs or iPSCs. When scrutinizing a table from a recent review (Martins-Taylor and Xu, 2012), which compares chromosomal macroscopic abnormalities between human ESCs (hESCs) and human iPSCs (hiPSCs) after prolonged culture, most alterations were common to both types of cells (i.e., trisomy of the X, 12, 8, 20q, and amplification of 17q, 20q11.21), while a minority was exclusively detected in either hESCs or hiPSCs. Such observations indicate that: (1) some genomic modifications recurrently (and clonally) seen must confer adaptive advantage to both cell types; (2) the reprogramming process is not responsible for a certain portion of the instability seen in hiPSCs (since hESCs, which have not been reprogrammed, also display some identical instability signatures); (3) some modifications could be linked to the specific pluripotent cellular type (i.e., trisomy 17, deletion of 18q12.1 to hESCs and deletion of 17q21.1 and 8q24.3 to hiPSCs, for instance); (4) other genomic alterations might not be observed due to their detrimental effect on pluripotency or survival of the cells [i.e., many aneuploidies, for instance, result in diminished fitness of cells or whole organisms (Sheltzer and Amon, 2011)].

Finally, some types of genomic modifications acquired during reprogramming or early passages have been reported to disappear, resulting in similar CNV levels among hESCs and hiPSCs at later passages (Hussein et al., 2011), indicating that CNV levels must be similar in both PSCs types. Reinforcing these findings, Taapken and coworkers (2011) had not observed differences in the incidence of abnormal karyotype (ESCs: 12.9% and iPSCs: 12.5%) when comparing 40 ESCs and 219 iPSCs lines from 29 different laboratories. Nonetheless, additional large-scale studies with ESCs and iPSCs cultured under equivalent conditions are needed to conclude if frequency and type of other genomic modifications differ in these two populations.

## The basis of aneuploidy in pluripotent stem cells

Several studies attempted to elucidate the molecular mechanisms causing imbalances in the chromosomal content of proliferative cells. Initially, the molecular mechanisms driving aneuploidy were explored in cancer cell lines, most likely due to the high frequency of chromosomal instability (CIN) associated with tumor phenotype. The “initial suspects” were molecules involved in the mitotic checkpoints. In fact, inactivation of mitotic checkpoints was shown to produce aneuploidy in different biological scenarios (Kops et al., 2005). It is more likely, however, that aneuploidy can arise by a combination of factors. For example, chromatid fragments can be the result from unrepaired DNA breaks. Chromosomes loss or gain can outcome from hypomethylation of centromeric DNA, defects in kinetochore or assembly proteins, as well as dysfunctional spindle and anaphase checkpoint genes (Yang et al., 2003; Iourov et al., 2006; Fenech et al., 2011).

Important tumor suppressors are central for many aspects of cell life, including control of proliferation, cell death, cell cycle arrest and chromosomal stability. P53 is one of the most studied tumor suppressors, and not surprisingly its pathway has been implicated in aneuploidy. The absence of P53 allows polyploid cells to proliferate and generate unstable aneuploid progeny (Holland and Cleveland, 2009; Talos and Moll, 2010). In ESCs, p53 maintains genetic stability through elimination of DNA-damaged ESCs from the replicative ESCs pool by directly suppressing the expression of transcription factor Nanog, which is necessary for pluripotency (Xu, 2005).

Another important gene for chromosomal stability is retinoblastoma (*Rb-1*). Stable inactivation of Rb leads to overexpression of mitotic arrest deficient 2 (Mad2), which induces aneuploidy (Hernando et al., 2004). Among Rb pathway's targets, changes in the expression of genes encoding proteins responsible for progression through mitosis, mitotic checkpoints and centrosome homeostasis, such as Plk1, Brca1, Aurora A/Stk6, Mad2 and Securin, were observed after Cre-mediated inactivation of Rb (Iovino et al., 2006). Little later, Iovino and colleagues demonstrated that Aurora A and Centrosome Protein A (CENP-A) might be necessary for the centrosome duplication, micronuclei formation and for the generation of aneuploidy induced by Rb-depletion (Amato et al., 2009a,b).

In 2010, independent studies confirmed the role of Rb in the regulation of CIN *in vivo* and in several cell types such as ESCs (Coschi et al., 2010; Manning et al., 2010; Van Harn et al., 2010), after observing a defective centromeric localization of Condensin II in Rb-depleted cells (Coschi et al., 2010; Manning et al., 2010). Altogether, these findings confirm that Rb plays a direct role in the maintenance of genomic stability by regulating the expression of specific genes important for chromosomes attachment to spindle, centrosome replication and checkpoints. Finally, Rb is critical, in haploinsufficiency, for the maintenance of chromosome stability in mouse embryonic stem cells (mESCs) (Zheng et al., 2002). Whether p53 and/or Rb deficiency is responsible for the natural aneuploidy observed in cultured stem cells (Peterson et al., 2011) remains to be determined.

In pluripotent cells, reduced time is dedicated to G phases, probably reflecting initial embryo demand for rapid cell accumulation (White and Dalton, 2005; Ruiz et al., 2011) before transcription is activated (Braude et al., 1988). The abbreviation of checkpoint-containing phases might propitiate aneuploidy.

Although the atypical S/M-predominant cell cycle is present in human pluripotent stem cells (hPSCs) (White and Dalton, 2005; Momcilovic et al., 2010), information on checkpoints' functionality is insufficient to draw any definitive conclusion (Becker et al., 2007; Mantel et al., 2007; Momcilovic et al., 2009, 2010). However, evidences from mESCs suggest checkpoint disruption is a feature of any highly proliferating cell with self-renewal capability, including hPSCs. At the G1 checkpoint, mESCs progress into S phase despite nucleotides depletion and DNA injuries (Aladjem et al., 1998; Hong and Stambrook, 2004), partially due to inefficient p53 nuclear translocation (Aladjem et al., 1998) and Chk2 sequestration in centrosomes (Hong and Stambrook, 2004).

Added to that, mitotic-spindle checkpoint, which helps to maintain chromosomal integrity during cell division, is uncoupled from apoptosis as microtubule-disrupted hESCs can progress through cell cycle even in the presence of mitotic-spindle-detached chromosomes (Mantel et al., 2007). Moreover, the decatenation checkpoint, responsible for preventing cell cycle progression in the presence of entangled chromosomes, is inefficient in mESCs causing severe aneuploidy (Damelin et al., 2005). Finally, mESCs self-renewal ability seems to depend on intrinsic nucleolin-mediated p53-pathway suppression (Yang et al., 2011). Since p53 pathway is essential for genomic integrity maintenance, self-renewable cells are naturally expected to exhibit some aneuploidy.

Given the unlimited nutrition at early development stages, generating new PSCs represents a better strategy than correcting damages to contain damage spread (Li and Huang, 2010). Accordingly, in cases in which genomic instabilities are excessively abundant or detrimental, cells might undergo differentiation (Ambartsumyan and Clark, 2008; Ruiz et al., 2011) or apoptosis (Momcilovic et al., 2010). Differentiation in developing embryos would restore cell cycle (Aladjem et al., 1998) and checkpoints (Damelin et al., 2005; Egozi et al., 2007; Mantel et al., 2007; Momcilovic et al., 2010) as well as induce many cells to exit cell cycle, selecting against propagation of most genome modifications. Alternatively, intrinsic PSCs loose checkpoints would allow some aneuploidies to persist during early development, while cells are still pluripotent.

## Concerns regarding aneuploidy for cell therapies

Cell therapy represents the most defiant and desired application envisioned for PSCs. While ESCs use would necessitate immunosuppressive treatment coupled to compatible matched cells from a donor bank, iPSCs would *in priori* exempt immunosuppressive treatment but, in the other hand, demand very precise gene-targeted correction when treating monogenetic diseases of known background.

First, aneuploidy represents a challenge for PSCs safety because the large amount of cells required for such applications is usually achieved by prolonged culture time. Long term cultivation favors aneuploidy (and other genetic modifications) in any cell type, and this has been clearly documented in PSCs (Enver et al., 2005; Maitra et al., 2005; Baker et al., 2007; Mayshar et al., 2010; Ross et al., 2011). Since pluripotent cells only exist transiently *in vivo*, giving rise to more committed cell lines following embryo development, forced maintenance of ESCs and iPSCs in the unnatural culture conditions for extended time is expected to allow accumulation of undesired and/or high level aneuploidy compromising their safety for cell therapy. Accordingly, cancer-related genes were identified in all chromosomes (12, 17, 20, X), whose imbalances are recurrently associated with culture adaptation (Draper et al., 2004; Maitra et al., 2005; Baker et al., 2007; Hovatta et al., 2010). Notably, teratomas from culture-adapted hESCs were described as more aggressive and less differentiated than teratomas from diploid hESCs (Herszfeld et al., 2006; Yang et al., 2008; Blum and Benvenisty, 2009). Moreover, as PSCs are teratogenic-prone, efficient differentiation protocols followed by very rigorous purification must be developed before cells are ready for human tests.

Since PSCs differentiation protocols are time-consuming and suboptimal, long-term culture of NPCs could be proposed as a solution to accelerate neurons availability. Nonetheless, continuous NPCs culturing increases chances of DNA changes accumulation as well as drive them to senescence. In fact, aneuploidy's increase after as short as 3 weeks could be detected in human cultured fetal NPCs (Yurov et al., 2005; Sareen et al., 2009). In one of these works (Sareen et al., 2009), trisomy of chromosomes 7 and 19 conferring growth advantage to the NPCs could be observed, representing a potential risk for tumor development following transplantation.

Indeed, an AT patient transplanted with fetal neural stem cells was diagnosed, 4 years later, with a brain tumor of non-host origin (Amariglio et al., 2009), highlighting the risks of stem cell “left-over” in differentiated cells to be used in humans. Although chromosomal stable fetuses seem to be selected for isolation of these cells, details of karyotype analyses after culture were absent. One could not discard transferred aneuploid neural stem cells as a source of tumor cells. In this regard, the paucity of data comparing aneuploidy levels among brain regions makes it even more difficult to predict the outcomes of applying neurons with aneuploidy acquired *in vitro* in therapy.

Concerning senescence, recurrent translocation of chromosome 1 q arm, previously associated with hematologic malignancies and pediatric brain tumors, allowed ESCs-derived NPCs to bypass it. However, such NPCs were unable to integrate into rat brains (Varela et al., 2012), reinforcing the negative impact of certain DNA changes on cell therapy. Finally, even though aneuploidy does not automatically imply in differentiation hurdles (Plaia et al., 2006), some were shown to hinder pluripotent cells differentiation to desired cell types. HESCs carrying deletion of 7q and an isochromosome 12 failed to express some markers of germ layers when differentiated into embryoid bodies (EB) and could not form teratomas *in vivo* (Imreh et al., 2006); ESCs with duplications of 1p32 and 1p36 were biased to differentiate to ectoderm, while their karyotypic normal counterparts tended to differentiate into mesoderm and endoderm (Yang et al., 2010), and trisomic 12 ESCs had enhanced potential to be differentiated in renal cells (Gertow et al., 2007). Detailed characterization of phenotypic changes related to proliferation, apoptosis signaling, immunogenicity and differentiation capacity conferred by all sets of aneuploidies are therefore mandatory before PSCs can be adopted for cell therapy.

## Aneuploid iPSCs and neural disease modeling

iPSCs offer the possibility to study diseases with genetic contribution for which the exacts DNA markers have not yet been established, taking advantage of somatic cells from diagnosed individuals. These cells are specially promising for the study of diseases displaying spectral phenotypes, in which each individual presents slightly different symptoms and treatment response patterns, complicating the choice of treatment. Finally, culture-dish format makes of iPSCs an extremely simple and convenient system to use. In spite of all these advantages, since PSCs have been proposed to exist mainly as aneuploid mosaics, whether aneuploid iPSCs will correctly reflect diseases unrelated to chromosomal abnormalities remains to be determined.

In this regard, certain types/proportion of aneuploidy should not represent an issue for studying central nervous system diseases, considering that mosaic aneuploidy is constitutive to the brain (Kingsbury et al., 2005, 2006; Rehen et al., 2005). Actually, aneuploidy seems to be a natural phenomenon during neurogenesis. In an attempt to elucidate whether aneuploidy takes place during neural fate patterning, Sartore et al. used both ESCs and iPSCs to recapitulate neurogenesis. Cells differentiated into NPCs and neurons exhibited heightened levels of aneuploidy after cellular commitment, especially chromosome loss, consistently with *in vivo* observations (Rehen et al., 2001; Kaushal et al., 2003; Westra et al., 2008). Also, displaced interactions between kinetochores and microtubules were hypothesized as a plausible driver of aneuploidy, since reduced levels of survivin, a protein participating in mitotic-spindle-microtubules anchorage to kinetochores and spindle assembly checkpoint (Lens et al., 2003; Saito et al., 2008; Sun et al., 2009), was found in NPCs (Sartore et al., 2011) together with previous findings of inefficient checkpoints (Damelin et al., 2005) and amplified centrosomes (Yang et al., 2003). The fact that aneuploidy has indeed been reported to increase during neuronal differentiation (Sartore et al., 2011) makes it rather a prerequisite for modeling diseases known to affect neurons.

Although information regarding iPSCs ploidy-status used to model diseases is usually absent or underestimated, no deficit in their differentiation capacity has been reported, and innumerous studies have achieved to recapitulate one or more features distinctive of pathological conditions using iPSCs (see below).

Corroborating their value as models and drug-testing platform, iPSCs-derived neurons from Rett's syndrome displayed MeCP2-decreased levels typical of the disease that could be restored following treatment with aminoglycoside antibiotics (Marchetto et al., 2010); iPSCs-derived neurons from patients with Alzheimer's disease showed elevated Aβ42–Aβ40 ratio, phospho-Tau and active GSK-3β, all reduced when treated with β- or γ-secretase inhibitor and modulator (Yagi et al., 2011; Israel et al., 2012); iPSCs-derived neurons from schizophrenic patients exhibited superior amounts of reactive oxygen species, reverted after valproic acid treatment (Paulsen et al., 2011) and reduced neuronal connectivity improved after loxapine administration (Brennand et al., 2011); motor neurons differentiated from spinal muscular atrophy's iPSCs had diminished expression of full length SMN-RNA, and the accumulation of SMN nuclear aggregates could be observed after exposure to valproic acid and tobramycin (Ebert et al., 2009). These studies demonstrate that, regardless of aneuploidy, iPSCs are capable to mirror at least some important aspects of chromosomal syndromes and adult diseases with genetic contribution. Furthermore, because different drugs were successfully used to revert disease hallmarks in iPSCs-derived models, their application for pharmacological screening is extremely promising.

Despite its indisputable value to achieve diseases better understanding and treatment, it is important to keep in mind that iPSCs display some limitations in modeling diseases. The first and more critical caveat relies on the fact that differentiation of iPSCs occurs in a two dimensional isolated environment, in the absence of diverse tissue-partners cells, structures and stimuli that may participate in a decisive way in disease establishment or maintenance. This would preclude the complete understanding of disorders and probably hamper high efficiency drugs development. Glial cells, for instance, have been suggested to participate in schizophrenia (Bernstein et al., 2009; Takahashi et al., 2011; Walterfang et al., 2011), as well as estrogen imbalance (Riecher-Rossler and Kulkarni, 2011). Likewise, late onset mental diseases are still difficult to depict using iPSCs (Yagi et al., 2011). Strategies such as accelerating the appearance of pathological phenotypes by the exposure to disease stimulator effects (i.e., oxidative stressors, hydrogen peroxide, MG-132 etc) as well as xenografting these human iPSC-derived diseased-cells to generate humanized animals might overcome these iPSCs limitations (Kunkanjanawan et al., 2011; Nguyen et al., 2011). Secondly, female hiPSCs poses an extra challenge. Despite female organism consists of a mosaic for X-inactivation, hiPSCs are clonal and all cells carry the same inactivated X-chromosome (Tchieu et al., 2010; Mekhoubad et al., 2012). Such limitation could be crucial for disease modeling specially for X-linked diseases.

Even if considering its limitations, iPSCs represents a practical system that simplifies diseases to fundamental aspects and complements biochemical and animal-based drug development by avoiding expenses with human-ineffective or toxic medicines.

## Conclusion

Low-level random aneuploidy (mainly chromosomal losses) has been described as a natural phenomenon in the human brain, however; because chromosomal losses and gains have been observed at higher frequencies in some brain pathologies, an aneuploidy frequency limit and/or chromosomal-specific aneuploidy type separating healthy from diseased brains seems to exist.

In order for iPSCs to be safely used as a cell repository for damaged or deteriorating tissue, as well as being able to modeling aspects of any given disease, they must retain the original tissue characteristics. Like PSCs, self-renewable NPCs also generate low-level aneuploidy in CNS. We believe chronic exposure to stressing environments related to complex predisposing genetic background could enhance aneuploidy generation in diseased-brains, which in turn could prevent defective cells clearance and participate in pathophysiology.

Aneuploidy's recurrent descriptions in PSCs raised the concern that these cells would not be appropriate for transplant or reliably model diseases. Observation that PSCs exhibit lax checkpoints and mostly exist as stochastic chromosomal mosaics, added to the fact that some normal tissues, including the brain, display this same pattern, suggest mosaic aneuploidy is intrinsic to these cells. Even more, both NPCs and PSCs share self-renewal capacity, suggesting random low-level aneuploidy could be linked to this characteristic. A stochastic process implies that random (advantageous, detrimental and neutral) aneuploidies must exist. However, in nature, diversity generation comes at a price: the same mechanism responsible for conferring organisms with more adaptability to adverse environment can generate dysfunction. Although many harmful chromosomal imbalances can direct cells to apoptosis, some could generate viable impaired cells resistant to cell death, resulting in biased differentiation capacities. Therefore, PSCs envisaged for transplant use should have their genome thoroughly examined for the presence of certain aneuploidies already associated to specific phenotypes in mosaic tissues and cancers. More studies need to be undertaken aiming comprehensive classification of pathogenic aneuploidies.

Mixing distinct proportions of aneuploid and euploid cells could also help to elucidate if a maximum aneuploidy limit is required to warrant healthy tissue physiology. In spite of its limitations, iPSCs still seem to provide a valid, easy to use, patient-specific model complementary to its animal and biochemical (purified receptor-based) counterparts, able to uncover aspects of poorly understood diseases and accelerate personalized and efficient drug choice/development. Moreover, provided these pluripotent cells are scrutinized for pathogenic-associated genomic instability, they could even serve in the future to treat neurodegenerative diseases in a cell-transplant manner.

### Conflict of interest statement

The authors declare that the research was conducted in the absence of any commercial or financial relationships that could be construed as a potential conflict of interest.
